# Development of microbial communities in biofilm and activated sludge in a hybrid reactor

**DOI:** 10.1038/s41598-022-16570-z

**Published:** 2022-07-22

**Authors:** Martyna Godzieba, Monika Zubrowska-Sudol, Justyna Walczak, Slawomir Ciesielski

**Affiliations:** 1 Department of Environmental Biotechnology, University of Warmia and Mazury in Olsztyn, Sloneczna 45G, 10-709 Olsztyn, Poland; 2 Department of Water Supply and Wastewater Treatment, Faculty of Building Services, Hydro and Environmental Engineering, Warsaw University of Technology, Nowowiejska 20, 00-653 Warsaw, Poland

**Keywords:** Ecological networks, Microbial ecology, Microbial communities

## Abstract

Microorganisms play a key role in biological wastewater treatment. The form in which biomass develops determines the efficiency and mechanisms of organic compound conversion, due to different conditions in various microbial structures. However, the results of studies comparing the microbial communities in biofilm and activated sludge have often conflicted. Therefore, this study compared the composition and development of the bacterial communities in biofilm and activated sludge in a hybrid reactor, employing 16S rRNA sequencing. Statistical analysis of the sequencing data included the identification of taxa characteristic to the biofilm and activated sludge, alpha and beta diversity analysis, and network analysis. These analyses indicated that the biofilm bacterial community was richer and more diverse than the activated sludge community. The mean numbers of OTU were 1614 in the biofilm and 993 in the activated sludge, and the mean values of the Chao1 (1735 vs. 1105) and Shannon (5.3 vs. 4.3) biodiversity indices were significantly higher for the biofilm. The biofilm was a better environment for development of nitrifiers (e.g., *Nitrosomonas*, *Nitrospira*) and phosphorus accumulating organisms (*Candidatus Accumulibacter*). Bacteria in the biofilm co-occurrence network had more connections (based on Spearman's rank correlation coefficient) with each other, indicating that they interact more than those in the activated sludge.

## Introduction

Hybrid biological reactors, which combine the growth of microorganisms in activated sludge and in biofilm, are widely used in wastewater treatment in a variety of different configurations. In the most common type of hybrid bioreactors, the biofilm develops on moving carriers added to an aeration tank, but there are also systems with other technological solutions like a rotating biological contactor or a submerged bed. Using bioreactors based on hybrid technology allows the concentration of biomass to increase and the efficiency of wastewater treatment to improve. An example of this type of technology is the Integrated Fixed-Film Activated Sludge-Moving-Bed Sequencing Batch Biofilm reactor (IFAS-MBSBBR), which is a modification of conventional sequencing batch reactor technology. In this reactor, both forms of biomass coexist in the same tank. The main advantages of this technology are the elimination of sludge bulking and the possibility of receiving a larger load of contaminants^1^.

Regardless of the technological solution used, microorganisms play a key role in biological wastewater treatment. The formation of the microbial community is influenced by many factors, including the operating conditions and the composition of the incoming wastewater^2^. An important factor affecting the efficiency of pollutant removal and the performance of the whole process is the sludge age. The sludge age, also called the solids retention time (SRT), is the time that the solid fraction (bacteria) spends in the reactor. Each group of bacteria has a different optimal time for multiplying, and too short a SRT leads to them being washed out of the system. The requirements of different bacteria in terms of the time needed for multiplication are quite different: a long SRT favours the development of nitrifying and filamentous bacteria, while short SRT favours phosphorus accumulating bacteria and denitrifies^3–5^.The form in which biomass develops also has a substantial effect on the final structure of the microbial community. The conditions in biofilm differ from those in activated sludge: for example, in biofilm there are concentration gradients of oxygen and nutrients, and less of these substances reaches the deeper layers of the biofilm. Therefore, activated sludge and biofilm have different mechanisms of pollutant removal^6^. An optimal thickness of biofilm is crucial for performance of wastewater treatment processes. If it is too thin, it does not provide anoxic conditions for the proliferation of denitrifying bacteria; if it is too thick, it is unfavourable to nitrifying bacteria, since it acts as a barrier that limits access to nutrients^7^.The form of a microbial community is also determined by its stage of development. In both activated sludge and biofilm, the proportions of individual groups of bacteria will change as the maturation process proceeds.

Advances in molecular techniques and next-generation sequencing have facilitated the study of complex bacterial communities in wastewater treatment systems. One of the most commonly used approaches in environmental microbiology studies is sequencing the 16S rRNA gene. By comparing the obtained sequences with sequences available in extensive databases, it is possible to identify bacteria present in environmental samples. 16S rRNA gene sequencing generates a vast amount of information on the entire microbial community and allows its taxonomic composition to be defined. However, this approach does not provide information on the active genes and metabolic pathways. Therefore to study gene expression and create a functional profile of microbial community it is required to use RNA based methods, such as metatranscriptomics. 16S rRNA gene sequencing enables estimation of the abundance of microorganisms forming a particular community but cannot provide information on the relationships between its members or the factors affecting its development^8^. One method for studying the interactions between microorganisms is creating a network representing the studied community, which allows it to be comprehensively analysed. The nodes in such networks symbolize operational taxonomic units (OTU). The nodes are linked to each other by edges, which represent the interactions between them (most often, a correlation in abundance). Visualization and analysis of the networks allows the key taxa in the studied communities to be determined, as well as potentially interdependent taxa, or those that potentially compete with each other.

Currently, several studies comparing biofilm and activated sludge communities have been published, but the results of these studies are often conflicting. For example, in some studies it was found that the biofilm and the activated sludge bacterial communities are similar, especially in mature forms of biomass^9^. Other studies, however, suggest the existence of significant differences in the structure of these two environments^10,11^. In studies by Jo et al.^12^, it was noted that certain groups of bacteria are common in both forms of biomass, while there are significant differences in their abundances, as well as in the interactions between community members, which was visible in differences in network topological features. Therefore, there is a need for a more in-depth study of these microbiomes, in particular their metabolic specialization in wastewater treatment and differences in their development and response to changing environmental conditions, e.g., aeration strategy, along with differences in interactions between community members. Therefore, the aim of this work was to characterize the differences in structure between the microbial community in the biofilm and the community in the activated sludge of a hybrid reactor treating municipal wastewater while using different aeration strategies. The role of particular bacterial species in organic compounds conversion in the studied reactor is discussed. The experiment was conducted over a long period of time, which allowed the two forms of biomass to be studied and compared at different stages of development. We used an approach involving a combination of 16S rRNA sequencing with analysis of microorganism co-occurrence networks. The sequencing technique provided information on the microbial composition of the communities in the biofilm and activated sludge. The second goal of this study was to obtain insight into the ecological relationships between members of these communities. This required statistical analysis of the obtained data and the creation of correlation matrices to quantify the co-occurrence of individual microorganism groups. Based on the correlation matrices, co-occurrence networks for the most strongly correlated taxa were created. The advantages of this approach are not only that the taxonomic composition of the studied environment was defined, but also that the groups of microorganisms that most often coexist and interact with each other were determined. Thus, this study provides new information on the ecology of bacteria in wastewater treatment systems and will help to develop understanding of the relationships between bacteria involved in wastewater compound transformations. Extending knowledge about these bacteria will enable better control of the pollutant removal processes in wastewater treatment systems.

## Results

### Bacterial community composition

**Figure 1:**
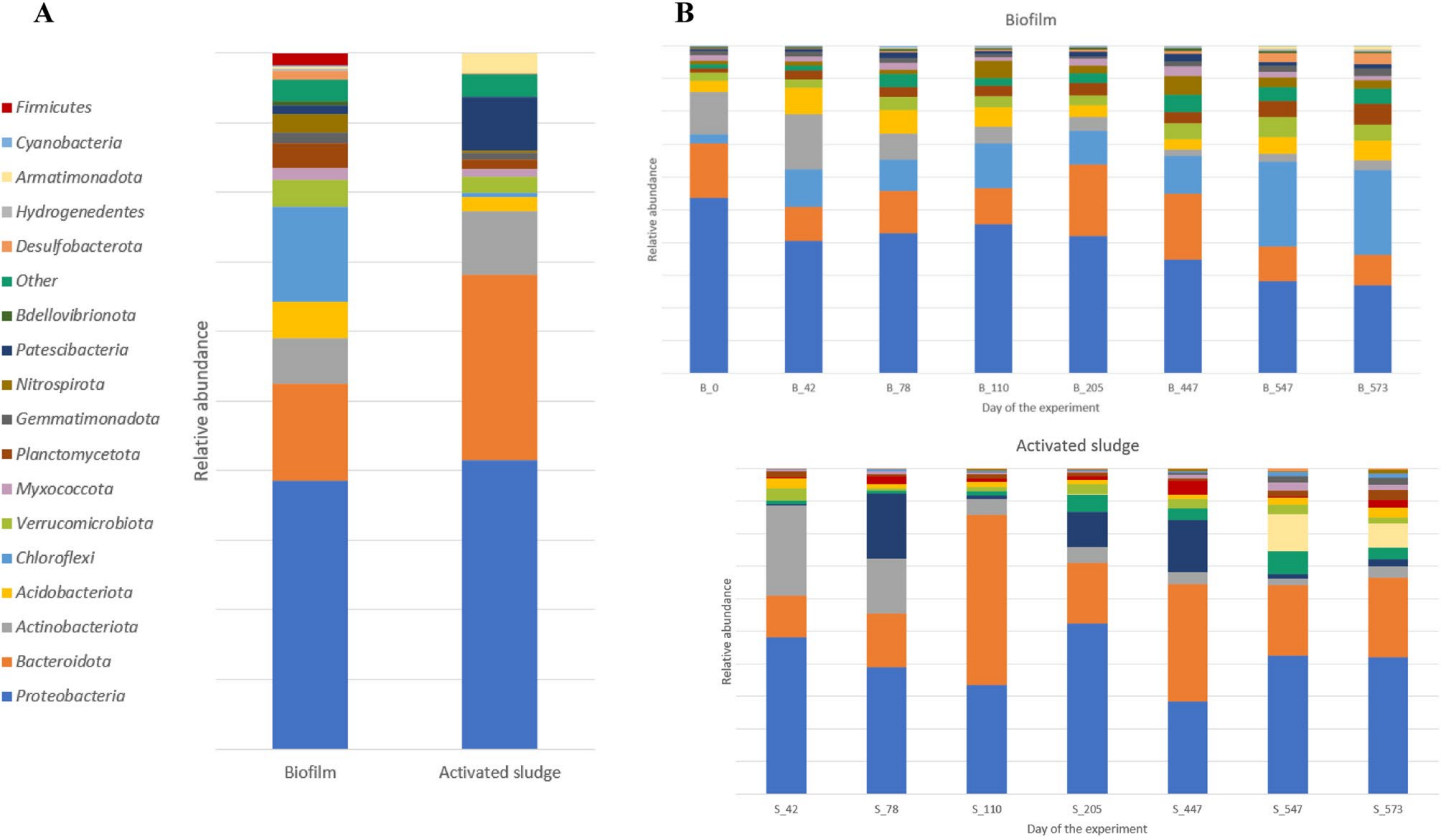
Relative abundance (%) of the most prevalent phyla in the biofilm and activated sludge samples in general, as the mean values of relative abundance from all biofilm and activated sludge samples (**A**), and in each individual sample (**B**). The graph shows only phyla which contributed more than 0.5% to the total bacterial community in at least one sample. The abundance of the remaining phyla was summed and labelled as "other".

**Figure 2:**
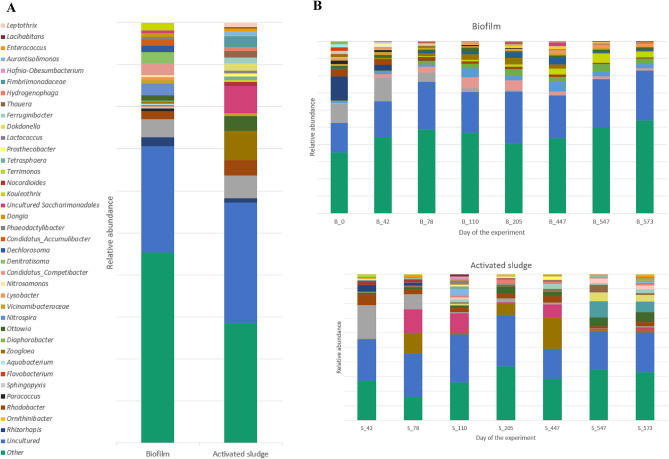
Relative abundance (%) of the most prevalent genera in the biofilm and activated sludge samples in general, as the mean values of relative abundance from all biofilm and activated sludge samples (**A**), and in each individual sample (**B**). The graph shows only genera which contributed more than 1.5% to the total bacterial community in at least one sample. The abundance of the remaining genera was summed and labelled as "other".

In order to study the microbial structure of the biofilm and activated sludge that were developing in the IFAS-MBSBBR reactor, a total of 15 samples were taken at intervals during an experiment lasting 573 days. The microbiome of both environments was described at the phylum and genus levels. A total of 26 bacterial phyla and 783 bacterial genera were identified. The most numerous phyla and genera in the biofim and activated sludge samples are presented in in Figs. 1 and 2. Both in the biofilm and the activated sludge, the most numerous phyla were *Proteobacteria*, with respective mean abundances of 39.3% ± 9.0 and 40.8% ± 8.2, and *Bacteroidota*, with respective mean abundances of 14.2% ± 4.9 and 26.1% ± 13.7. Additionally, the phylum *Chloroflexi* was rather abundant in the biofilm (with a mean abundance of 13.9 ± 8.1), while *Actinobacteriota* and *Patescibacteria* were relatively abundant in the activated sludge (with mean abundances of 9.0% ± 9.6 and 7.5% ± 8.1, respectively). STAMP analysis identified significant overrepresentations of *Chloroflexi*, *Acidobacteriota,* and *Nitrospirota* in biofilm and of *Firmicutes* in activated sludge.

In both environments, the abundances of various groups of bacteria changed over time. In the biofilm, the abundance of *Proteobacteria* and *Actinobacteria* gradually decreased, while that of *Chloroflexi* increased. In the activated sludge, the changes in abundance were larger and more rapid, and the abundance of *Bacteroidota* changed to the largest extent, ranging from 12.7% after 42 days of reactor operation to 52.3% after 110 days, when it was the predominant phylum. The abundance of *Patescibacteria* also changed substantially: its abundance was highest on the 78th, 205th and 447th days of the process, reaching values of 20.1%, 11.0%, and 7.2%, respectively. Similar changes took place in the abundance of *Armatimonadota*, which reached 11.4% and 7.6% on the 547th and 573th day, but did not exceed 0.1% in the samples taken at other times.

At the genus level, the less abundant genera (each < 1.5% of the total bacterial community) combined to constitute the largest shares in all samples of biofilm and activated sludge samples (mean abundance of 45.6% ± 5.8 and 30.5% ± 6.0, respectively). Initially, *Ornithinibacter* was relatively abundant in the biofilm, which is the reason for its fairly large mean abundance of 4.3% ± 5.3%. Over time, however, the abundance of this group decreased substantially, and at the end of the process, it was only 0.3%. Similarly, the abundance of *Rhizorhapis* was 13.92% in the first sample, but then it decreased and this genus was not detected after the 205th day. The changes in the abundance of *Nitrospira* and *Candidatus Competibacter* are also noteworthy, first increasing and then decreasing. *Nitrospira* was most abundant in the sample from 447th day (5.7%), and *Candidatus Competibacter*, in the sample from 110th day (6.4%). The abundance of the remaining genera did not exceed 5% at any time during this study.

**Figure 3:**
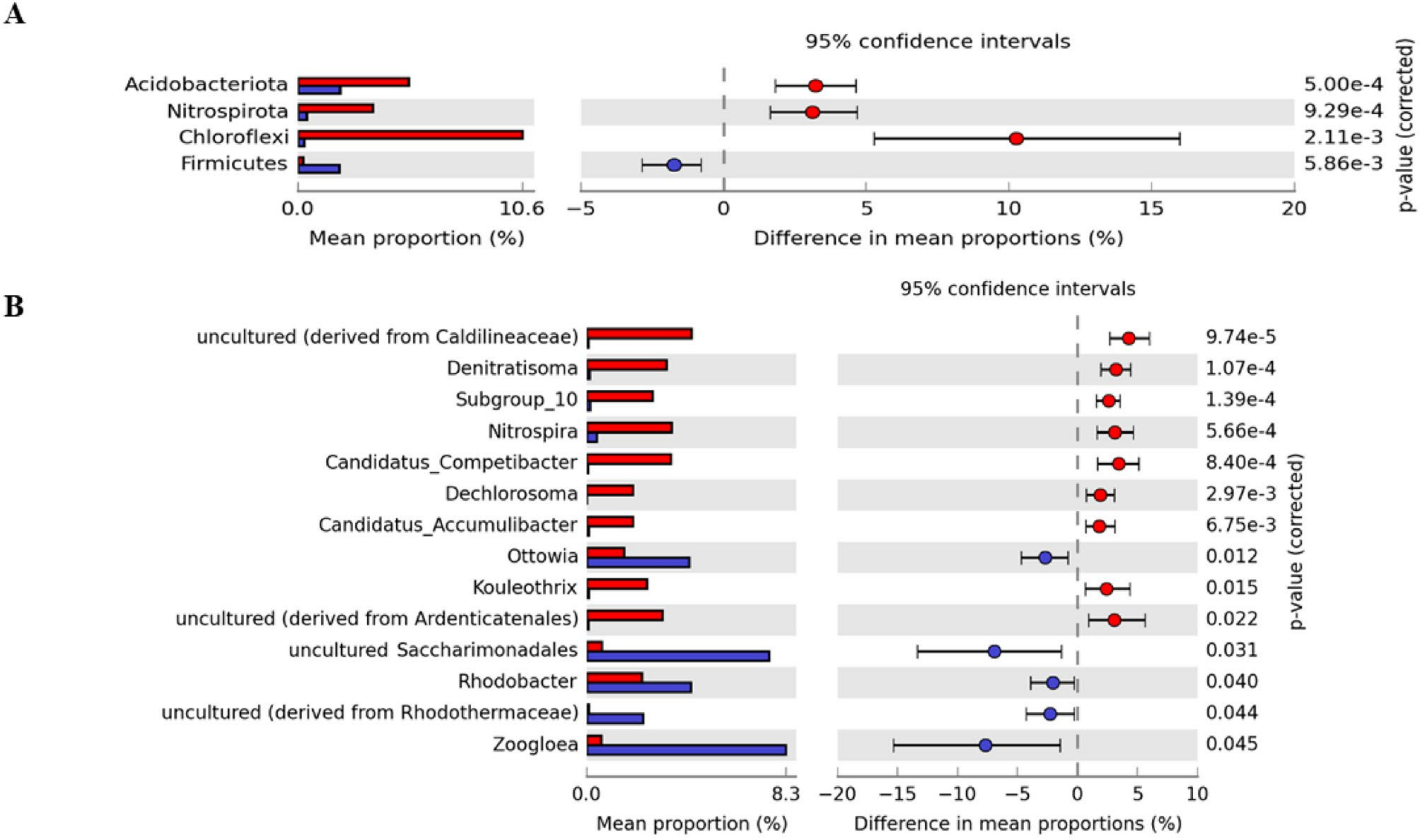
Mean proportions of microbial phyla (**A**) and genera (**B**) that differed significantly between biofilm (red) and sludge (blue) samples. Plots were made using Statistical Analysis of Metagenomic Profiles (STAMP) software. *P*-values and confidence intervals were calculated with White’s nonparametric t-test.

In the samples of activated sludge, the abundance of *Ornithinibacter* also decreased significantly at the beginning of the experiment (from 23.0% in the first sample and 12.5% on the 78th day to values below 3% in subsequent periods). Generally, the abundances of individual genera changed more rapidly in the activated sludge than in the biofilm. There were also rapid decreases and increases in the abundance of many groups of bacteria in the following periods, particularly in the case of uncultured *Saccharimonadales* and *Zoogloea*. Figure 3 shows groups of bacteria that differed significantly between biofilm and sludge samples. *Denitratisoma*, *Nitrospira*, *Candidatus Competibacter*, *Dechlorosoma*, *Candidatus Accumulibactrer*, and *Kouleothrix* were significantly more abundant in the biofilm than in the biomass, while *Zooglea*, uncultured *Saccharimonadales*, *Rhodobacter*, and *Ottowia* were significantly less abundant in the biofilm.

### Bacterial diversity

Bacterial community indices were estimated using the EZBioCloud platform (Table 1). The average Good’s coverage of all samples was 99.75% ± 0.047%, indicating that the sequencing coverage was very high. The total number of OTUs differed between samples and types of biomass. The mean number of OTUs was 1614 ± 141 for biofilm and 993 ± 109 for activated sludge. The Chao1 index was used to evaluate community richness, i.e., the number of species in the biofilm and activated sludge communities, and the Shannon index was used to measure community diversity, taking into account both the abundance and the evenness of the species. The mean values of these indices indicated that the biofilm community was richer and more diverse than the activated sludge community (Chao1: 1734.64 ± 138.59 vs. 1105.72 ± 138.59; Shannon: 5.34 ± 0.23 vs. 4.27 ± 0.41). The differences between communities were all statistically significant (*P* < 0.05).

**Table 1:** Estimates of diversity and richness indices for biofilm and activated sludge samples.

Type of biomass	Sample Name	OTUs	Chao1	Shannon	Good's coverage of library (%)
Biofilm	B_0	1380	1538.80	4.87	99.63
	B_42	1567	1707.56	5.18	99.73
	B_78	1710	1836.02	5.51	99.72
	B_110	1660	1780.21	5.36	99.74
	B_205	1563	1652.45	5.35	99.79
	B_447	1557	1676.54	5.42	99.76
	B_547	1606	1685.47	5.43	99.76
	B_573	1869	2000.10	5.62	99.71
Mean		1614	1734.64	5.34	99.73
Activated sludge	S_42	1026	1121.76	3.69	99.74
	S_78	962	1088.88	3.90	99.79
	S_110	910	1008.95	4.09	99.81
	S_205	875	985.94	4.20	99.82
	S_447	911	1037.90	4.74	99.78
	S_547	1162	1280.44	4.55	99.72
	S_573	1108	1216.18	4.70	99.73
Mean		993	1105.72	4.27	99.77

**Figure 4:**
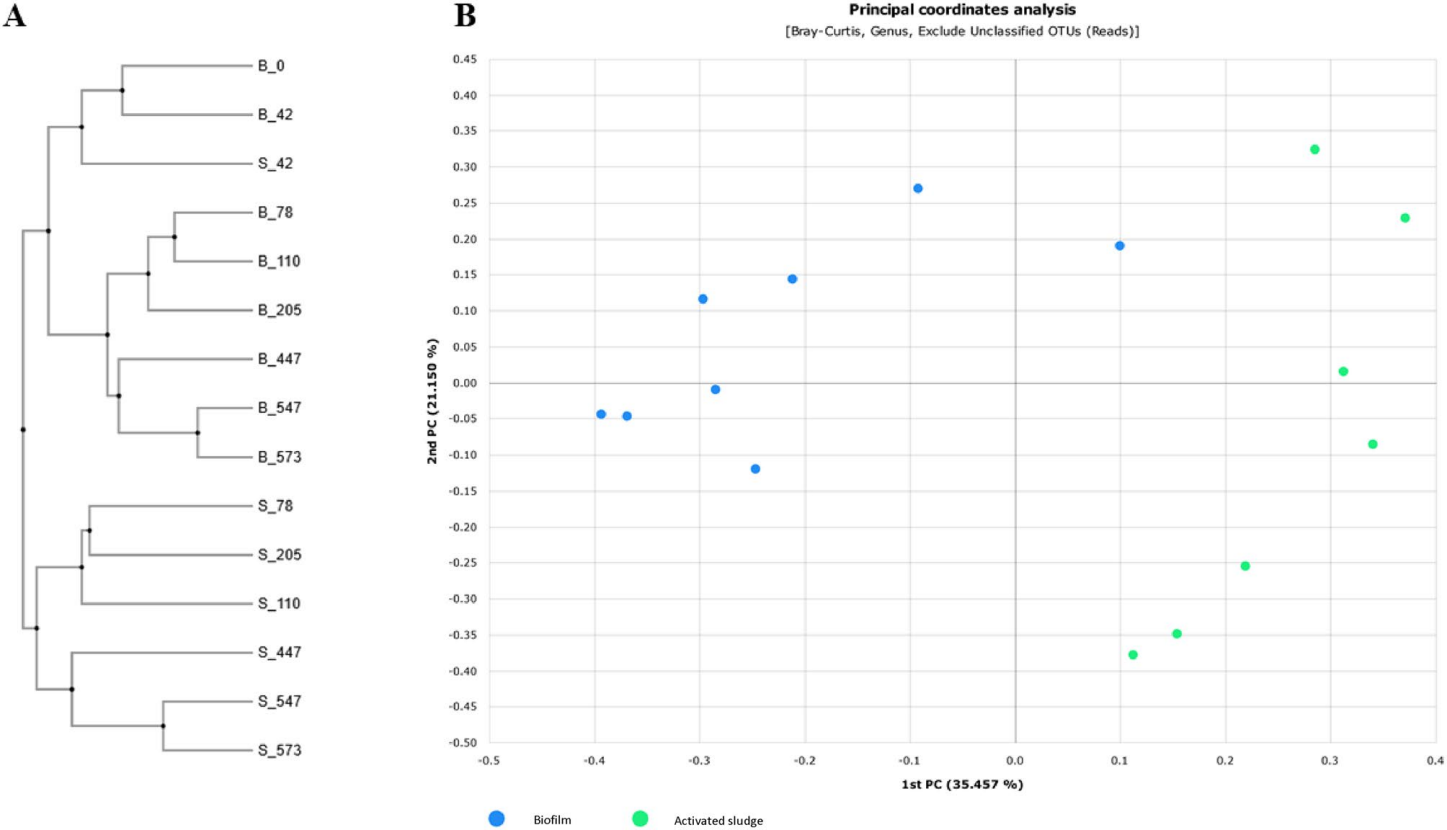
Hierarchical clustering and Principal Coordinates Analysis plot of biofilm (B) and sludge (S) samples.

Figure 4 shows the results of beta diversity analysis based on the Bray–Curtis dissimilarity. Principal Coordinates Analysis showed that the biofilm and activated sludge samples grouped into two separate clusters, although the distances between individual samples were quite large. Hierarchical analysis indicated the development of biofilm and activated sludge was independent and confirmed the distance of the differences between these two types of biomass.

**Figure 5:**
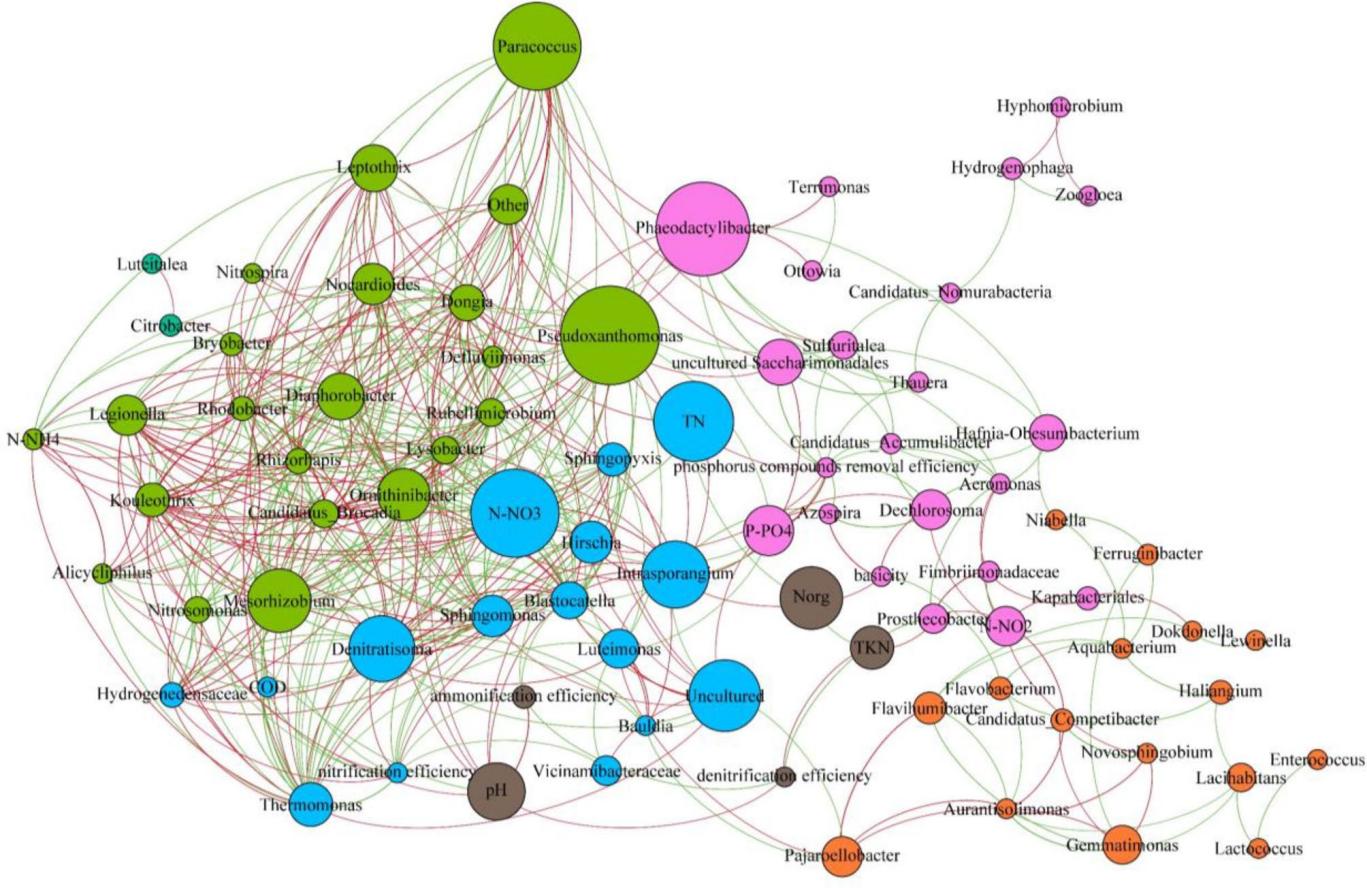
Network of the biofilm microbiome with nodes representing taxa at the genus level or efficiencies of pollutant removal and edges representing correlations (green edges—positive correlation; red edges—negative correlation).

**Figure 6:**
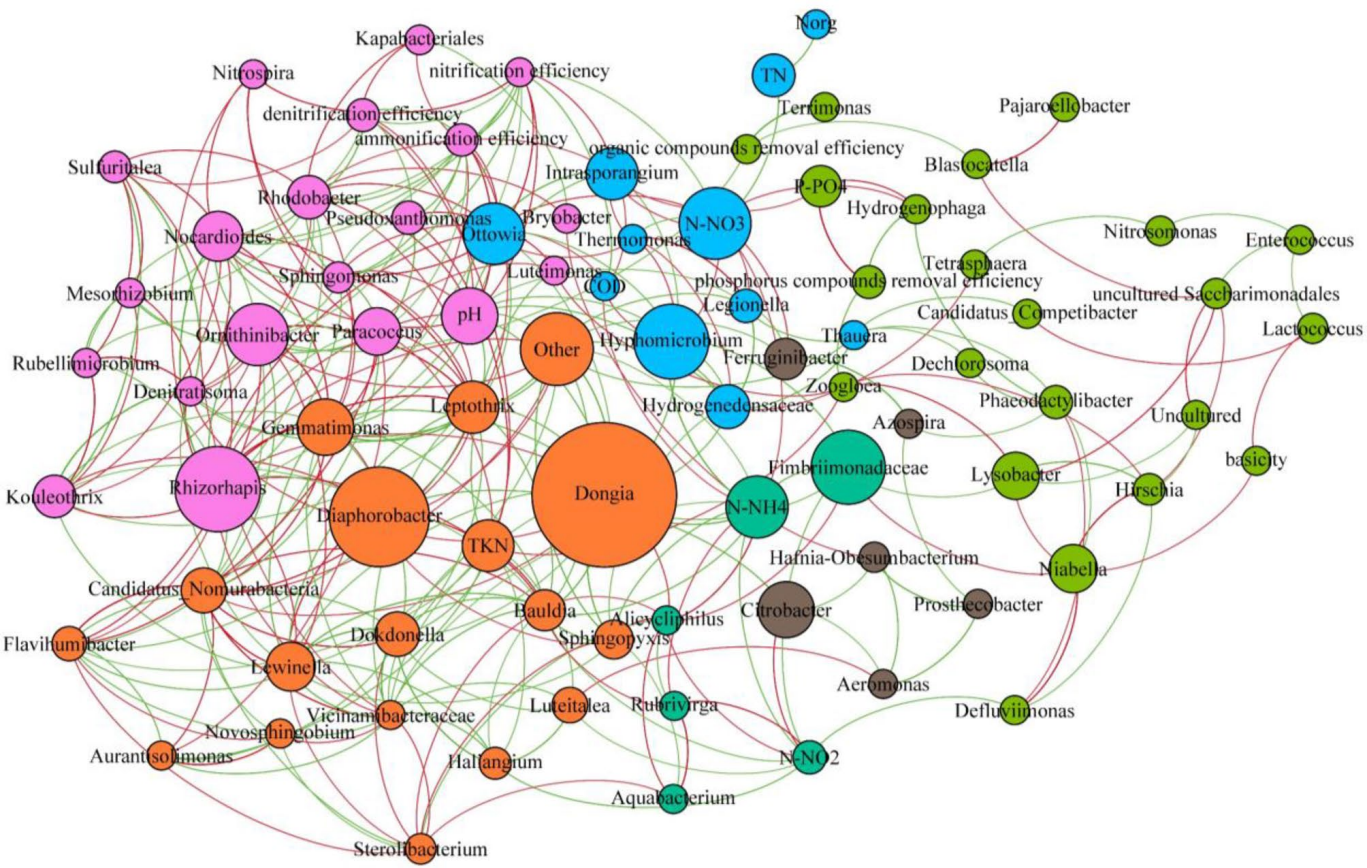
Network of the activated sludge microbiome with nodes representing taxa at the genus level or efficiencies of pollutant removal and edges representing correlations (green edges—positive correlation, red edges—negative correlation).

To model interactions and relationships between bacteria in the biofilm and activated sludge, co-occurrence network analysis was used. In the present study, two networks were created that represent the co-occurrence of genera in the biofilm and activated sludge. In Figs. 5 and 6, the color of each node is based on its modularity class parameter, and its size is based on its betweenness centrality. The basic parameters characterizing both networks are presented in Table S1. In general, the biofilm network had more connections between nodes than the activated sludge network, and the distance between nodes was smaller in the biofilm network, indicating that the microorganisms creating the biofilm are more closely related and have more relationships between them. Both networks had the same number of nodes (83), but the biofilm network had more edges (connectors between nodes symbolizing co-occurrence). In both networks, the number of positive associations was slightly higher than that of negative associations, accounting for 55% of the total number of connections. The mean clustering coefficient (i.e., the ratio between the observed and the maximum possible number of links between a node and its neighbors) was higher for the biofilm than for the activated sludge (0.556 vs. 0.432). Similarly, the network density, which is the ratio between the observed number edges and the maximum possible number of them, was higher for the biofilm (0.073 vs. 0.05). The network diameter (the distance between the two most distant nodes) was shorter for the biofilm than for the biomass (6 vs. 7). Likewise, the average path length, which is the number of edges in the shortest path between pair of nodes, was shorter in the biofilm network than in the activated sludge network (1.984 vs. 2.241). The mean node degree (the number of edges between one node and other nodes in the network) was greater in the biofilm network than in the activated sludge network (6.012 vs. 4.12). Node degree ranged from 1 to 31 in the biofilm network and from 1 to 23 in the activated sludge network. In the biofilm network there were four nodes with the highest degrees (≥ 30) that can be considered hub nodes: *Diaphorobacter*, *Rhizorapis*, *Mesorhizobium*, and *Pseudoxanthomonas*. These microorganisms had 61.5% positive and 38.5% negative connections with other microorganisms. Interestingly, although the abundance of *Mesorhizobium* and *Pseudoxanthomonas* was low (not exceeding 1.5% of the total bacterial community in any sample) they had positive associations with highly abundant bacteria, e.g., *Ornithinibacter*. The activated sludge network also had 4 hub nodes (with node degree ≥ 20): *Nocardioides*, *Gemmatimonas*, *Leptothrix* and *Rhizorhapis*. These hub nodes were connected to other nodes by similar amounts of positive and negative edges (51.8% and 48.2%, respectively). The size of the nodes in the created networks is proportional to their betweenness centrality (a parameter that indicates the frequency of occurrence of a particular node on the paths between two other nodes). High values of betweenness centrality indicate that a node has a central location in a network, while low values indicate that it has a peripheral location^13^. Microorganisms with high betweenness centrality play key roles and act like bridges between other bacteria in the network. In the biofilm network, *Paracoccus*, *Phaeodactylibacter*, and *Pseudoxanthomonas* had the highest values of betweenness centrality, whereas in the activated sludge network, *Dongia*, *Diaphorobacter*, and *Rhizorhapis* had the highest values.

The networks were constructed with additional nodes representing the efficiency of pollutant removal processes, i.e., removal of organic and phosphorus compounds, as well as denitrification, ammonification, and nitrification. In the biofilm network, the efficiencies of phosphorus compound removal and of nitrification had the most associations with microbial nodes (9 positive and 2 negative, and 6 positive and 4 negative, respectively). The efficiency of phosphorus removal was positively associated with the abundance of *Candidatus Accumulibacter*, *Dechlorosoma*, *Thauera*, and uncultured *Saccharimonadales*, while nitrification was positively associated with the abundance of taxa such as *Nitrosomonas*, *Sphingomonas*, and *Thermomonas*. The remaining efficiencies of pollutant removal had no or only 1–2 connections with microbial nodes. In the activated sludge network, in contrast, all efficiency nodes were connected with those of microbes, with degree ranging from 2 to 9. The nitrification and ammonification efficiency nodes had the highest degree and were positively associated with, for example, *Nocardioides*, *Rhodobacter*, and *Sphingomonas*. Organic compound removal efficiency was positively associated with *Blastocatella*, *Ornithinibacter*, and *Terrimonas*, whereas in the biofilm network, it had no edges.

## Discussion

The biodiversity of both forms of biomass was examined using the Chao1 and Shannon indices. In all samples, the values of both these indices were significantly higher in the biofilm. This finding is consistent with the results obtained by Dong et al.^14^ with a similar reactor; however, Jo et al.^12^ obtained contrary results and did not observe similar differences. They speculated that this lack of differences might be caused by the fact that both flocks and biofilm result from microbial aggregation. On the other hand, biofilms have a layered structure and the conditions in each layer are a bit different, which is optimal for development of different groups of bacteria. This might be the reason why, in the present study, the biofilm community was richer and more diverse than that of the activated sludge.

Various aeration strategies were applied during the experiment, which influenced the growth of the microorganisms in the reactor. Apparently, the change in aeration strategy had a stronger effect on the activated sludge bacterial community than on the biofilm community. In PCoA analysis, the biofilm samples, except for the first sample, were clustered more tightly than the activated sludge samples. This finding indicates that the biofilm community is more resistant to changing environmental conditions than the activated sludge community. This resistance to change can be explained by the fact that biofilm is a complex bacterial structure and is surrounded by a layer of extracellular polymeric substances (EPS) produced by the microbes, which acts as a physical barrier protecting the bacterial cells from environmental stress.

In both types of biomass, *Proteobacteria* and *Bacteroidota* were the most abundant phyla. Proteobacteria are typically the most abundant group in bacterial communities in municipal wastewater treatment systems. This taxon has many subgroups, the most common of which is *Betaproteobacteria*, which are largely responsible for removal of organic matter and nutrients^15^. The next most abundant phyla in the biofilm were *Chloroflexi*, *Actinobacteriota* and *Acidobacteriota*. *Chloroflexi* are filamentous bacteria that favor the formation of microbiological structures. Their filaments protrude from flocs and biofilm, which probably gives them better access to substrates in the surrounding liquid^9^ and is believed to provide scaffolds on which activated sludge flocs form^16,17^. *Actinobacteriota*, as well as *Proteobacteria* and *Bacteroidota*, have been reported to be some of the core phyla that are widespread in wastewater treatment systems^18,19^. *Acidobacteriota* are involved in P-removal and have the potential to utilize various organic compounds, including glucose, xylose, acetate, and fatty acids^20^. After *Proteobacteria* and *Bacteroidota*, the next most abundant phyla in the activated sludge were *Actinobacteriota* and *Patescibacteria* (especially the uncultured *Saccharimonadales*). *Patescibacteria* have a small cell size and a reduced genome, which suggests that they are host-dependent syntrophs or parasitic^21^. Their small cell size might be advantageous in oligotrophic environments, due to their correspondingly increased surface-to-volume ratio^22^. The genus *Zooglea* was more abundant in activated sludge than in biofilm. *Zooglea* produce exopolysaccharides that participate in the formation of activated sludge flocs^23^.

Network analysis revealed that positive associations between the abundance of taxa and the efficiency of nitrogen and phosphorus compound removal were more common in the biofilm community than in the activated sludge community. This may indicate that biofilm is a better environment for the growth of bacteria capable of decomposing these compounds. This hypothesis is also supported by the high abundance of certain groups of bacteria in the biofilm. For example, both the phylum *Nitrospirota* and the genus *Nitrospira* were significantly more abundant in the biofilm than in the activated sludge. *Nitrospirota* includes bacteria that oxidize nitrites to nitrates (NOB), as well as the recently discovered comammox bacteria (complete ammonia oxidizers), which can perform both steps of the nitrification process^24,25^. It could suggest that in the biofilm the commamox process could be important in ammonia oxidation. *Nitrosomonas*, a representative of ammonia oxidizing bacteria (AOB), was also more abundant in the biofilm, but it was not as numerous as *Nitrospira*. The results of this study are similar to those of Shao et al.^26^, who compared attached biofilm and activated sludge flocs in an Integrated Fixed-Film Activated Sludge—Sequencing Batch Biofilm Reactor (IFAS-SBR). *Nitrosomonas* had also negative association with anammox bacterium *Candidatus Brocadia*, which may be caused by the competition for the same substrate. Probably *Candidatus Brocadia* was abundant in deeper layers of a biofilm, where concentration of oxygen is low. Hosokawa et al.^27^ reported that anammox bacteria occur together with *Patescibacteria*, however in our study this taxa was more abundant in the activated sludge. Perhaps this is an example of the cooperation of biofilm with activated sludge in a hybrid system. In the biofilm, denitrifiers were highly abundant, although they were also quite common in the activated sludge. From this group of microorganisms, *Denitratisoma*, *Rhodobacter* and *Dechlorosoma* were present in the biofilm, while *Rhodobacter* and *Thaurea* were present in the activated sludge.

Bai et al.^28^ observed similar results and concluded that biofilm is a better environment than activated sludge for the development of *Denitratisoma*, *Rhodobacter* and *Dechlorosoma*, due to the longer solid retention time associated with biofilm. Interestingly, those authors also observed that there were more phosphorus accumulating organisms (PAO) in the activated sludge. This differs from the results of the present study, in which, for example, *C. Accumulibacter*, a PAO, was more abundant in the biofilm than in the activated sludge flocs. McIlroy et al.^29^ reported that *Candidatus Competibacter* was also quite numerous in biofilm samples; this is a glycogen-accumulating organism that is believed to compete for resources with PAOs. Although associations between bacteria and efficiency of different pollutant removal were observed, there is no clear connection between bacteria and concentration of nitrogen forms. For instance, since *Nitrosomonas* oxidize ammonia, it can be expected that in the network, abundance of *Nitrosomonas* will be inversely proportional to the ammonia concentration and directly proportional to the nitrates concentration. However, such obvious dependencies did not occur in the analyzed network. The reason for this may be that in the tested system, different processes, carried out by many different groups of microorganisms, and affecting the concentration of individual forms of nitrogen, take place. Because many different groups of bacteria cooperate with each other in removing nitrogen compounds from wastewater, creating a kind of functional whole, it is difficult to determine the contribution of individual units in this process.

Although the number of nodes was similar in the biofilm and activated sludge networks, the number of interactions between nodes was greater in the biofilm network. These results indicate that the genera present in the biofilm interact with each other to a greater degree than those in the activated sludge. The correlations in the abundance of taxa were predominately positive in both types of biomass. A greater number of positive than negative correlations was also noted by Jo et al.^12^ in their research, but the number of positive associations in their study (92% in the biofilm network and 75% in the activated sludge network) was much higher than in ours (55%). Based on their betweenness centrality, key taxa were identified in both networks. Microorganisms with high betweenness centrality often lie on the shortest path between two other nodes, and for this reason, they are considered important for the flow of information between community members^30^. One of these key taxa in the biofilm network was *Paracoccus*, which is a heterotrophic nitrifying and aerobic denitrifying bacterium that can also remove phosphorus^31^. Other groups of bacteria with high betweenness centrality were *Pseudoxanthomonas* (capable of removing nitrogen and phosphorous under aerobic conditions^32^ and *Phaeodactylibacter* (a denitrifier^33^). In the activated sludge network *Dongia* and *Diaphorobacter* had the highest betweenness centrality; these taxa are involved in the transformation of nitrogen compounds^27,34^. The differences between the biofilm and the activated sludge network also indicate that other bacteria may be responsible for the transformation of nitrogen compounds in these two environments. Network analysis and the high values of betweenness centrality of the mentioned above taxa suggest that even taxa with a low abundance might play important roles in the bacterial community. The role of such bacteria in wastewater treatment processes is not well known and requires further investigation.

## Conclusions

This study compared the structures of the microbial communities in biofilm and activated sludge from an IFAS-MBSBBR hybrid reactor. The bacterial composition of the biofilm differed from that of the activated sludge, although some core groups of bacteria (*Proteobacteria*, *Bacteroidota*, *Actinobacteriota* and *Acidobacteriota*) were highly abundant in both types of biomass. The biofilm was more diverse in terms of bacterial composition and a better environment for the development of nitrifiers (e.g., *Nitrospira*, *Nitrosomonas*) and phosphorus accumulating organisms (*C. Accumulibacter*). The bacteria in the biofilm network had more connections with each other than those in the activated sludge network. Furthermore, in the biofilm network, more bacteria were connected with nitrogen and phosphorus removal efficiency, which indicates that biofilm might play a larger role in the removal of these pollutants. Network analysis also revealed that even bacteria with low abundance might play important roles in the community, although these roles require further investigation. A better understanding of the contribution of these taxa will provide a more complete picture of these complex communities and the relationships between the bacteria that create them.

## Materials and methods

### Description of the IFAS-MBSBBR reactor and operating conditions

The study was conducted in a laboratory model of a sequencing batch reactor with an active volume of 28 L in which microorganisms developed in the form of activated sludge, and biofilm on EvU-Perl moving bed (Integrated Fixed-Film Activated Sludge—Moving-Bed Sequencing Batch Biofilm Reactor—IFAS-MBSBBR). The cylindrical carriers with dimensions of Φ5 mm, h = 8 mm, and specific surface area of 600 m^2^/m^3^ constituted 25% of the active volume of the reactor. The concentration of activated sludge was maintained at a level of approximately 1.7 g MLSS/L. The operation of IFAS-MBSBBR was fully automated and controlled through DreamSpark Premium software (SCADA system). Wastewater was supplied to the reactor by means of a peristaltic pump Ismatec Ecoline, and its content was stirred with a slow-speed paddle mixer CAT R-50D. Oxygen concentration was measured by an optical probe Oxymax COS61D cooperating with a transmitter Liguiline CM 442. The system operated in an air-conditioned room, ensuring 20̊C temperature in the reactor.

The reactor was supplied with synthetic wastewater simulating the composition of municipal wastewater. The following wastewater characteristics were assumed: COD 510 mg O_2_/L, TN 60 mg N/L, N-NH_4_^+^ 40 mg N-NH_4_^+^/L, P-PO_4_^3−^ 8 mg P-PO_4_^3−^/L, pH 7.7. Their preparation employed peptone (135 mg/L), starch (45 mg/L), glucose (45 mg/L), glycerine (0.0495 ml/L), ammonium acetate (225 mg/L), NaHCO_3_ (125 mg/L), Na_2_HPO_4_ (15 mg/L), and KH_2_PO_4_ (4.5 mg/L).

The reactor operated in a system of 3 eight-hour cycles per day. A single treatment cycle involved the following phases: I phase without aeration with wastewater dosing (50 min), I phase with aeration (190 min), II phase without aeration with wastewater dosing (30 min), II aerobic phase (150 min), sedimentation (50 min), decantation (10 min). During 573 days of the experiment duration, periods with blower unit turned off were introduced in aerobic phases to obtain an intermittent aeration strategy, or oxygen concentration was changed. Moreover, between the 447 and 547th research day, the contamination load of the reactor was decreased through a decrease in wastewater dose from 10 L/d to 6.6 L/d (Table 2). Samples of infow and outfow water were collected between December 2018 and June 2020. Chemical analysis was carried out according to standard methods (Table_S2).

**Table 2:** Reactor operating conditions.

Period (days)	Oxygen concentration (mg O_2_/L)	Aeration strategy	Duration of periods with blower unit turned on (t_withAer._) and off (t_withoutAer._) during aerobic phases for intermittent aeration t_withAer._/ t_withoutAer._ (min.)	Organic Loading Rate (g COD/m^3^d)
0–42	3.0	Continuous	–	540
43–78	3.0	Intermittent	40/10	540
79–110	2.0	Intermittent	30/10	540
111–205	1.5	Intermittent	30/10	540
206–447	1.5	Intermittent	20/10	540
448–547	1.5	Intermittent	20/10	360
548–573	1.5	Intermittent	20/10	540

### DNA extraction

Biomass samples for microbiological tests were collected from biofilm and activated sludge at specified intervals from December 2018 to June 2020. The samples were stored at -25° C. DNA was isolated from 200 ng of biomass (both activated sludge and biofilm) using a FastDNA™ SPIN Kit for Soil (MP Biomedicals, USA). The isolation procedure was performed according to the manufacturer's instructions. A Qubit fluorometer (Invitrogen, USA) was used to measure the amount of isolated DNA. The obtained DNA was stored at − 18 °C until further analysis**.**

### High-throughput 16S rRNA gene sequencing

High-throughput Illumina sequencing targeting the V3-V4 region of the 16S rRNA gene was performed with S-d-Bact-0341-b-S-17 and S-d-Bact-0785-a-A-21 primers^35^ and NEBNext®High-Fidelity 2X PCR Master Mix (Bio Labs inc., USA) following the manufacturer’s manual. The sequencing reactions were carried out with a MiSeq sequencer and a MiSeq Reagent Kit V2 (Illumina, USA) by applying paired-end technology with read lengths of 2 × 250 bp following the manufacturer’s protocols.

### Sequencing data analysis

Raw paired-end sequences were processed using the QIIMEII^36^ software package. Paired-end sequences were merged using the fast-join algorithm. Reads that software could not merge were excluded from further analyses. The filtering process quality score (q < 20) was obtained using the Cutadapt algorithm^37^. Chimeric sequences were detected and excluded from analyses using USEARCH^38^.16S rRNA OTUs were picked from the Illumina reads using a closed-reference OTU picking protocol against the SILVA_V_138 database^39^. Sequences were clustered at 97% identity and trimmed to span only the 16S rRNA V4 region flanked by the sequencing primers. Taxonomy assignments were associated with OTUs based on the taxonomy associated with the SILVA_V_138 reference sequence defining each OTU.

### Statistical analysis

Statistical comparison of biofilm and activated sludge samples was made using STAMP software (Statistical Analysis of Metagenomics Profiles (http://kiwi.cs.dal.ca/Software/STAMP)) ^40^. Significance was determined with White’s non-parametric t-test. Results with *P* < 0.05 were considered significant.

Bacteria co-occurrence networks were created based on a correlation analysis of the taxonomic profiles^41^. For the analysis, the most abundant taxa in the studied metagenomes were selected. Spearman's correlation analysis (with a significance threshold of α = 0.05) was carried out using STATISTICA v.13.1 (StatSoft, Inc, Tulsa, OK, USA). Networks were plotted only for strongly correlated taxa (with correlation coefficients higher than 0.75 or lower than -0.75). The correlation matrices obtained in this way were used to create networks using Gephi software^42^. Alpha and beta diversity analysis were performed with the use of EZBioCloud platform^43^.

## Supplementary Information

Supplementary Table S1.

Supplementary Table S2.

## Data Availability

The sequences reads were deposited in the NCBI Sequence Read Archive (SRA) under the accession number PRJNA793374.
