# Supplementary material for: Development of microbial communities in biofilm and activated sludge in a hybrid reactor
Source: Sci Rep. 2022 Jul 22;12:12558. doi: 10.1038/s41598-022-16570-z (PMC9307651; doi:10.1038/s41598-022-16570-z)
Supplement: Supplementary file 1 — Supplementary Table S1. [file 41598_2022_16570_MOESM1_ESM.docx]

**Development of microbial communities in biofilm and activated sludge in a hybrid reactor**

Martyna Godzieba^[1](#Affiliation_1)^* , Monika Zubrowska-Sudol^[2](#Affiliation_2)^ , Justyna Walczak^[2](#Affiliation_2)^ , Slawomir Ciesielski^[1](#Affiliation_1)^

^1^University of Warmia and Mazury in Olsztyn, Department of Environmental Biotechnology,Sloneczna 45G, 10-709 Olsztyn, Poland

^2^Warsaw University of Technology, Faculty of Building Services, Hydro and Environmental Engineering, Department of Water Supply and Wastewater Treatment, Nowowiejska 20, 00-653 Warsaw, Poland

* Corresponding author. E -mail address: [martyna.godzieba@uwm.edu.pl](mailto:martyna.godzieba@uwm.edu.pl)

**Table S1.** Biofilm and activated sludge network statistics

|  | Biofilm network | Activated sludge network |
| --- | --- | --- |
| Number of nodes | 83 | 83 |
| Number of edges | 499 | 342 |
| Positive correlations | 275 | 190 |
| Negative correlations | 224 | 152 |
| Network density | 0.073 | 0.05 |
| Modularity | 0.306 | 0.416 |
| Average degree | 6.012 | 4.12 |
| Average clustering coefficient | 0.556 | 0.432 |
| Average path length | 1.984 | 2.241 |
| Network diameter | 6 | 7 |
